# A mannogalactoglucan from steam-exploded *Hericium erinaceus*: structural elucidation, digestion resistance and gut microbiota-modulating prebiotic activity

**DOI:** 10.1016/j.fochx.2026.104233

**Published:** 2026-07-21

**Authors:** Shuang Chen, Qing Yan, Liping Liu, Jie Feng, Qing Kong, Jingsong Zhang, Jie Geng, Jinyan Wang, Qingbin Guo, Yanfang Liu

**Affiliations:** a Institute of Edible Fungi, Shanghai Academy of Agricultural Sciences, National Engineering Research Center of Edible Fungi, Key Laboratory of Edible Fungi Resources and Utilization (South), Ministry of Agriculture and Rural Affairs, Shanghai 201403, China; b State Key Laboratory of Food Nutrition and Safety, College of Food Science and Engineering, Tianjin University of Science and Technology, Tianjin 300457, China; c Shandong Medicine Technician College, Taian, Shandong 271000, China

**Keywords:** *Hericium erinaceus*, Steam explosion, Structural characterization, *In vitro* digestion and fermentation, Gut microbiota, Short-chain fatty acid

## Abstract

Steam explosion (SE) pretreatment effectively enhanced the extraction yield and bioactivity of polysaccharides from *Hericium erinaceus* (*H. erinaceus*), demonstrating notable therapeutic potential. In this study, a polysaccharide fraction (Q60E) was isolated from SE-treated *H. erinaceus*. Structural analysis revealed that Q60E (*M*_*w*_, 8.89 × 10^4^ g/mol) was a mannogalactoglucan, featuring a backbone of →3)-*α*-Man*p*-(1→, →6)-*β*-Glc*p*-(1→, →3,6)-*β*-Glc*p*-(1→, →3)-*α*-Glc*p*-(1 → and →4,6)-*β*-Gal*p*-(1 → linkages with side chains of →4)-*α*-Glc*p*-(1 → and terminal *β*-Glc*p*-(1 → residues. Based on the shape factor *ρ* (1.71) and the Mark-Houwink-Sakurada parameter (exponent *α*, 0.51), Q60E adopted a random coil conformation in aqueous solution. *In vitro* fermentation studies demonstrated that Q60E modulated gut microbiota by promoting beneficial genera (*Lactobacillus*, *Lachnospira* and *Bifidobacterium*) while suppressing pathogenic *Fusobacterium*. Furthermore, Q60E fermentation additionally enhanced the acetic acid and total SCFAs production, underscoring its prebiotic capacity. These findings highlight the potential of the mannogalactoglucan from SE-pretreated *H. erinaceus* as effective prebiotics for gut health.

## Introduction

In China, *Hericium erinaceus* (*H. erinaceus*) is a traditional delicious and medicinal mushroom (Friedman, 2015). As one of the most important functional components, polysaccharides have demonstrated potential in preventing diseases such as gastric ulcer, diabetes mellitus, hyperlipidemia and neurodegenerative disorders, all of which were closely linked to intestinal health (Cheng et al., 2016; Lee & Hong, 2010; Shang et al., 2015). Increasing evidence suggested that gut microbiota (GM) dysbiosis is a key underlying factor in these conditions, which has spurred growing interest in polysaccharide-based strategies for modulating the GM (Lee & Hong, 2010; Shang et al., 2015; Chen et al., 2025). Although, the active polysaccharides in *H. erinaceus* possessed high medicinal value, their low yield remained a major limiting factor for further development.

To overcome the limitation of low polysaccharide yield, a more efficient wall-breaking technology - steam explosion (SE) pretreatment, can be introduced. SE pretreatment converted insoluble cell wall components of the raw material into soluble fractions, thereby improving the extraction rate of active constituents (Sui et al., 2018). The underlying principle involved utilizing high-temperature saturated steam to generate elevated pressure within the tissue pores of the material, followed by an instantaneous pressure release. This process created micropores and cracks on the cell wall surface, which facilitated the release of soluble compounds, such as polysaccharides, from the cell wall matrix (Liu et al., 2021). Our previous study found that SE pretreatment markedly increased the yield of crude polysaccharides from *H. erinaceus* with values from 8.43% to 29.08%. Furthermore, SE pretreatment lowered the molecular weight (*M*_*w*_) and generated low *M*_*w*_ fractions, which could also improve bioactivity by modulating GM composition, highlighting its considerable potential for further development (Chen et al., 2024).

We also demonstrated that SE not only significantly enhanced the extraction yield of water-soluble polysaccharides from *H. erinaceus*, but also modified the branching ratio of the glucan main chains while reducing their *M*_*w*_ (Chen et al., 2025). In addition, these SE-induced changes in *M*_*w*_ and branching ratio subsequently influenced the distribution of GM and the increase in the production of metabolites, such as acetic acid and total acid (Chen et al., 2025). During the isolation and purification of the crude polysaccharide fractions from *H. erinaceus* fruiting bodies, a relatively homogeneous polysaccharide component with low *M*_*w*_ was also found to be released under SE pretreatment. To date, most reported polysaccharides from *H. erinaceus* fruiting bodies have been characterized as glucans or galactoglucans, with backbones primarily composed of →4)-Glc*p*-(1→, →6)-Gal*p*-(1→, →6)-Glc*p*-(1→, or →3,6)-Glc*p*-(1 → linkages, occasionally accompanied by small amounts of mannose and fucose (He et al., 2017; Liu et al., 2020a). However, the structural characteristics of this polysaccharide obtained by SE pretreatment have not yet been reported and its potential bioactivity in modulating GM remains to be elucidated.

The current study extends previous investigations into the effects of SE pretreatment on the glycosidic linkage patterns of polysaccharides derived from *H. erinaceus.* To systematically elucidate the influence of SE on polysaccharide structure and its subsequent role in GM modulation, we hypothesize that SE treatment releases a low-molecular-weight mannogalactoglucan (Q60E) that resists upper gastrointestinal digestion, reaches the colon intact, and selectively enriches beneficial bacterial genera while enhancing short-chain fatty acid (SCFA) production. To test this hypothesis, we conducted a comprehensive structural characterization of Q60E, complemented by 16S rRNA gene-based microbial community analysis and quantification of key microbial metabolites (SCFAs). These findings could highlight the potential utility of this polysaccharide as a targeted prebiotic agent capable of promoting SCFA production.

## Materials and methods

### Materials

The fruiting body of *H. erinaceus* was supplied from Shanghai Peiyuan Agricultural Development Co., Ltd. A suite of reagents, including digestive enzymes (pepsin, trypsin), bile salt, deuterated dimethyl sulfoxide (DMSO), heavy water (D_2_O), methyl iodide, sodium borohydride and standard monosaccharides, were acquired from Sigma-Aldrich (St. Louis, USA). Starch and bicinchoninic acid (BCA) protein testing kit was procured from Solarbio (Beijing, China). The standard of SCFAs and 98% Vitamin K1 were procured from Macklin (Shanghai, China). All other compounds employed in the current research were of analytical grade.

### Extraction and isolation of polysaccharides from *H. erinaceus*

The crude polysaccharide component in *H. erinaceus* pretreated by SE was extracted and prepared by hot water combined with 75% ethanol precipitation according to the pre*v*iously reported method (Chen et al., 2024). The freeze-dried crude polysaccharide component was re-dissolved to produce a 20 mg/mL solution. Ethanol was then added slowly to achieve a concentration of 45% (*v*/v), followed by precipitation at 4 °C for 12 h and centrifugation (8000 *g*, 15 min) to remove the large *M*_*w*_ fractions. Subsequently, additional ethanol was incrementally introduced to the supernatant until the concentration attained 60% (v/v). After 12 h of ethanol precipitation at 4 °C, the second precipitation was acquired *via* centrifugation (8000 *g*, 15 min), washed with 60% ethanol at the same concentration, then dissolved, volatilized and freeze-dried to obtain the second fraction named Q60E. The yield of Q60E was determined through dividing the mass of freeze-dried relatively homogeneous polysaccharides fraction by the mass of *H. erinaceus* crude polysaccharides. The corresponding formula for calculating was as follows:

$$Y_{1}\left(\%\right)=\frac{M_{1}}{M}\times 100$$

***Y*₁**: Relative yield of the polysaccharide fraction (%), ***M*₁**: The mass of freeze-dried polysaccharide fraction (g), ***M***: The mass of crude polysaccharides (g).

### Compositional analysis and structural characterization

#### Analysis of chemical composition

Quantification of carbohydrates in the Q60E sample was performed as described by Dubois et al. (1951) using the phenol‑sulfuric acid approach. The protein quantification was employed with a BCA assay kit (Cortes-Rios et al., 2020).

#### Monosaccharide composition analysis

In a sealed V-shaped vial, the polysaccharide sample of Q60E (2.0 mg) was hydrolyzed for 4 h at 110 °C using 3 mL of 2 mol/L trifluoroacetic acid (TFA). After hydrolysis, the TFA was evaporated under an air stream, followed by the addition of 3 mL methanol and further blow-drying. Repeated the above operation for 5 times until TFA was completely evaporated. The hydrolysate was dissolved with ultrapure water, and then fixed into a 50 mL volumetric flask. The monosaccharide composition and corresponding molar ratios in the hydrolysate were determined by high-performance anion-exchange chromatography (HPAEC) (Liu et al., 2022).

#### Fourier transform infrared (FT-IR) spectroscopy

The Q60E polysaccharide was analyzed using an IS50 FT-IR spectrometer. Spectra were recorded over the wavenumber range of 4000–400 cm^−1^, with 16 scans at a resolution of 4 cm^−1^.

#### Homogeneity, *M*_*w*_ distribution and conformation analysis

The Q60E component was dissolved in distilled water to obtain a 5 mg/mL solution and then filtered across a 0.45 μm aqueous microporous membrane for later use. Its homogeneity and average *M*_*w*_ were measured *via* HPSEC coupled with UV and refractive index (RI) detectors, a multi-angle laser scattering detector (MALLS, for direct *M*_*w*_ measurement), and a DP viscometer (for viscosity). The analytical column consisted of a protective column (TSKPW_XL_, Japan), TSKgel PW_XL_4000 and PW_XL_6000 (7.8 mm × 300 mm) in series. The eluent consisted of 0.05 mol/L NaNO_3_ buffer, flowing at 0.5 mL/min under 35 °C column temperature. The MALLS detector operated at a wavelength of 623.8 nm. Data and conformational parameters including weight average *M*_*w*_, number average molecular weight (*M*_*n*_), polydispersity index (PDI = *M*_*w*_ / *M*_*n*_), intrinsic viscosity ([*η*]), radius of gyration (*R*_*g*_) and hydrodynamic radius (*R*_*h*_) were gathered and evaluated utilizing Astra software (Version 6.1.1, Wyatt Tech. Santa Barbara, CA, USA).

#### Methylation analysis

The methylation analysis procedure of Q60E was carried out following the methodology outlined by previous studies (Liu et al., 2018). Firstly, the dried polysaccharide sample of Q60E (2 mg) was irradiated under an infrared lamp for 4 h, and then 0.5 mL of DMSO was added to the bottle and stirred magnetically at 85 °C for 2 h until the polysaccharide sample was completely dissolved. Then 20 mg of dried and ground NaOH powder was added into the bottle, and it was dissolved by magnetic stirring at room temperature for 3 h. Subsequently, 0.5 mL of methyl iodide was introduced and stirred at 30 °C in the absence of light for 2.5 h, which was converted into partially methylated glycol acetate through a series of methylation, hydrolysis, reduction, acetylation and the partially methylated glycol acetate (PMAA) was analyzed by gas chromatography–mass spectrometry (GC–MS) system fitted with the DB-5MS column (30 m × 0.25 mm × 0.25 μm). The peak positions and linkages of PMAAs were determined by the relative retention durations of ion fragments, and the proportion of methylated sugars was computed based on the peak area.

#### Nuclear magnetic resonance (NMR) analysis

The Q60E polysaccharide (25 mg) was repeatedly (3×) dissolved in 0.6 mL of D_2_O and then freeze-dried. After sufficient dissolution, the sample was centrifuged (12,000 *g*, 20 min), with the supernatant subsequently transferred to an NMR tube. Finally, one-dimensional (1D) nuclear magnetic resonance (^1^H NMR and ^13^C NMR) and two-dimensional (2D) nuclear magnetic resonance (COSY, TOCSY, HSQC, HMBC and NOESY) spectra were recorded at 70 °C by employing Bruker VNMRS 600 NMR spectrometer (Yao et al., 2021; Chen et al., 2025).

### *In vitro* digestion

The three-stage process of simulating the *in vitro* digestion of Q60E polysaccharide solution was conducted in accordance with the previously documented approach (Chen et al., 2024). Before the experiment, electrolyte solutions of saliva (SSF), gastric (SGF) and intestinal fluid (SIF) for digestion were prepared in advance. Overall, 3.2 mL of SSF containing 150 U/mL *α-*amylase was added to a test tube containing 4 mL of Q60E polysaccharide sample and kept at 37 °C for 2 min. The digestion process continued by adding 3.2 mL SGF (containing 2000 U/mL pepsin) to the mixture, adjusting the pH to 3, and maintaining at 37 °C for 4 h. Subsequently, 3.2 mL of SIF containing 100 U/mL trypsin and 10 mM bile salts were introduced to the digestive juice. The pH was adjusted to 7.0, and the system was incubated at 37 °C for 6 h. The digestion reaction solution taken out at each time point was soaked in boiling water for 15 min to stop enzymatic activity, and the supernatant after centrifugation (5000 *g*, 10 min) was tested for subsequent indicators.

### *In vitro* human feces fermentation

The fermentation experiment was conducted following established methods for preparing basic medium and fecal suspension reported in previous research (Abdin et al., 2025; Chen et al., 2024). Briefly, fresh human fecal specimens were obtained from six healthy volunteers (three males and three females, aged 22–28) who maintained a balanced diet for a period of three months and did not take any antibiotics or other medications. Equal volumes of fecal suspension from each donor were pooled to obtain a representati*v*e inoculum, thereby minimizing inter-individual variation. This study utilized fecal samples collected from six healthy donors as the sole human-derived material, and these samples were pooled to prepare a mixed fecal suspension. All collection procedures were approved and exempted by the Institutional Ethics Committee of the Shanghai Academy of Agricultural Sciences, as the study involved solely the use of fecal samples from healthy donors without any direct human experimentation. Therefore, no specific approval code was assigned to this study. All donors were informed of the purpose and procedures of the study and provided written informed consent for the use of their fecal samples. The study protocol complied with the institutional ethical standards, and no foreseeable harm or discomfort was associated with sample collection. Based on preliminary experiments, the optimal concentration ratio for mixed fermentation was determined by evaluating the levels of metabolites, such as SCFAs, produced during fermentation. A basal medium without any carbon source was blank control (CON), 1% (*w*/*v*) starch constituted the primary carbon source group (AmY), and the combination of 0.2% Q60E fraction and 0.8% starch was designated as the experimental group (Q60E).

Subsequently, 9.0 mL of medium containing the experimental samples was mixed with 1.0 mL of a 10% (w/v) fecal suspension. The mixture was then subjected to anaerobic fermentation at 37 °C for 0, 6, 12, 24 and 48 h. Fermentation broth was collected at each time point for subsequent analysis.

### The physicochemical properties analysis of simulated digestion and fermentation

After simulating digestion and fermentation, the contents of reducing sugar and total sugar were evaluated by 3,5- dinitrosalicylic acid (DNS) and phenol sulfuric acid methods (Dubois et al., 1951; Miller, 1959), respectively. The changes of *M*_*w*_ in three digestion stages were measured by HPSEC. The pH value in the fermentation process was tested with a standard pH meter.

### The determination of short-chain fatty acids (SCFAs)

The extraction and preparation of SCFAs during each period of simulated *in vitro* fermentation, the quantification of standard samples (acetic, propionic, n-butyric, i-butyric and n-valeric acids) and the testing methods by gas chromatography-flame ionization detector (GC-FID) were all referenced from previously reported research (Chen et al., 2025).

### Microbial composition analysis

Following 48 h of fermentation, the effect of polysaccharide on GM was studied by high-throughput sequencing method. The three experimental of CON, AmY and Q60E were used to complete DNA extraction using the QiagenQIAamp Fast DNA Fecal Mini-Kit. 1% agarose gel electrophoresis was employed to determine DNA extracted from fermentation samples. PCR amplification was conducted with TransStart Fastpfu DNA Polymerase in 3 parallels per sample. The PCR results from the identical sample were combined and analyzed using 2% agarose gel electrophoresis. The amplicon was purified using an AxyPrepDNA gel recovery kit, with DNA concentration determined by QuantiFluor™-ST Blue Fluorescence Quantification System (Promega). The bacterial 16S rRNA V3-V4 hypervariable regions were subsequently amplified with 338F-806R primer pairs and followed by Illumina Miseq sequencing. The sequence data generated in this study have been deposited in the NCBI SRA under BioProject accession number PRJNA1464037.

### Statistical analysis

All experiments were carried out independently for three times, and the results were expressed as mean ± standard deviation (SD). Statistical significance was analyzed using SPSS 27 software. For SCFAs, total/reducing sugar and pH data, one-way ANOVA combined with Tukey's post-hoc test, and the significance threshold was *p* < 0.05. Graphic data were plotted using Origin software version 2025 (OriginLab Corporation, USA). Microbial sequence analysis employed the DADA2/deblur denoising algorithm to generate amplified sequence variants (ASVs) and abundance data. For Shannon and Simpson indices, the non-parametric Kruskal-Wallis test was used to detect overall differences among groups, followed by Dunn's multiple comparison test with false discovery rate (FDR) correction. LEfSe analysis was performed on the Majorbio Cloud Platform using an All-against-all multi-group comparison strategy. Prior to analysis, count data were normalized by total sum scaling (TSS) to relative abundance proportions across all samples. Unclassified or unknown taxa at any taxonomic level were removed before analysis. All data processing was carried out on the Majorbio cloud platform (www.cloud.majorbio).

## Results and discussion

### Yield and chemical composition analysis

**Fig. 1:**
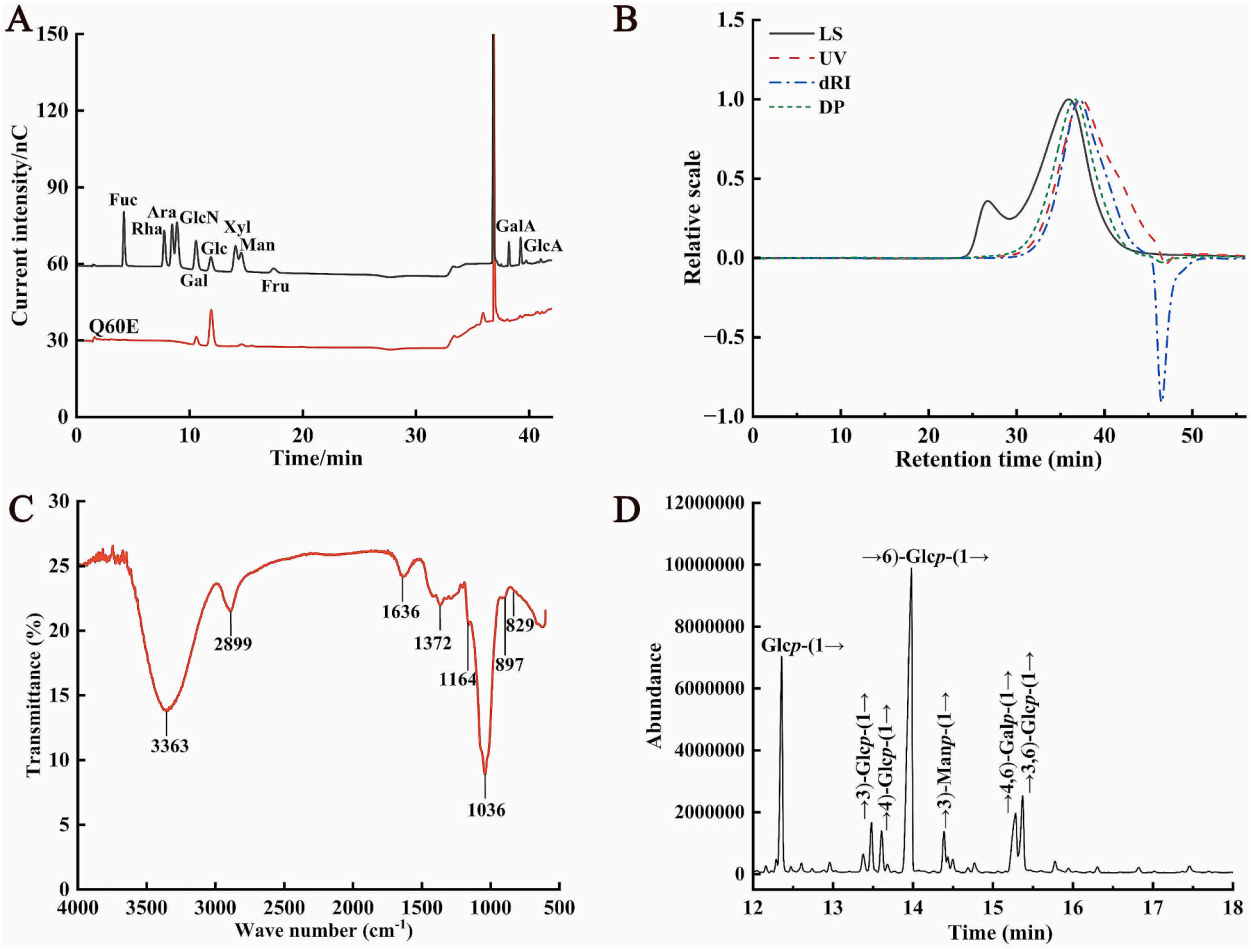
The monosaccharide compositions (A), the elution profiles of polysaccharide fraction (B), FT-IR spectra (C) and total ion chromatogram (D) of Q60E from the *H. erinaceus* by SE pretreatment*.*

It was reported that polysaccharide components with different *M*_*w*_ ranges could be separated and isolated by fractional ethanol precipitation combined with ethanol washing, which was simple and efficient (Liu et al., 2018). In this study, a relatively homogeneous fraction, designated as Q60E, was successfully isolated through systematic optimization of the initial polysaccharide solution concentrations and the gradient ranges employed in graded ethanol precipitation. The relative yield of Q60E was 19.21% (*w*/w) based on the weight of crude polysaccharides. In addition, the polysaccharide sample contained 89.71% total sugars and 5.37% protein. The monosaccharide composition analysis of Q60E was summarized in Table S1 and Fig. 1A, which mainly consisted of galactose (8.35%), glucose (87.14%) and mannose (4.51%), with a calculated molar ratio of 1.9: 19.3: 1.0. The notably high abundance of glucose indicated that glucan was likely the dominant structural skeleton of this polysaccharide fraction.

### The *M*_*w*_ and conformational characteristic

It has been proved that HPSEC combined with multi-detectors (section 2.3.4) was a tool to systematically characterize the molecular and conformational characteristics of macromolecular polysaccharide, and the chromatograms of Q60E was shown in Fig. 1B. A single symmetric peak was observed in both the RI and UV profiles, confirming that Q60E was a polysaccharide fraction with uniform relative molecular weight distribution. Based on previous studies, this observation suggested that the protein fraction might not be covalently attached to the sugar residues at the periphery of the molecule, but rather potentially linked to the polysaccharide backbone (Kang et al., 2012). Future purification using ion-exchange or affinity chromatography could further separate minor components such as proteins, thereby complementing the characterization of this fraction. In addition, the *M*_*w*_ and *M*_*n*_ of Q60E were 8.89 × 10^4^ g/mol and 5.37 × 10^4^ g/mol, respectively, and the PDI was calculated to be 1.66. This observation might be attributed to variations in branching degree and chain length of the polysaccharide, with the PDI value exhibiting a positive correlation with chain length (Shi et al., 2019). Notably, the natural polysaccharides exhibiting monodisperse characteristics generally possess a narrow PDI range of 1.50–2.00 (Guo et al., 2020). Q60E exhibited a relatively narrow polydispersity (PDI = 1.66) and a single symmetric peak in the HPSEC-MALLS-RI profile, indicating that it was the dominant fraction within this molecular weight range. The conformational parameters including the *R*_*g*_ and *R*_*h*_ were determined to be 15.80 nm and 9.26 nm, respectively. The chain architecture and conformation of a specific polymer in solution determine the ratio of the radius of gyration to the hydrodynamic radius (*ρ* = *R*_*g*_/*R*_*h*_) (Wang & Cui, 2005). According to reports (Guo et al., 2020; Shi et al., 2019), *ρ* was greater than 2.0 for an extended rigid chain, 0.7–0.8 for a homogeneous sphere, and 1.3–1.8 for a linear flexible random coil chain. The *ρ* value of Q60E was 1.71 in Table S2, indicating that it had a random coil chain conformation in aqueous solution. Furthermore, the Q60E exhibited an intrinsic viscosity ([*η*] = 15.81 mL/g). Based on the data of conformational characteristic in HPSEC (Table S2), the Mark-Houwink-Sakurada equation of Q60E was:

$$\left[\eta \right]=4.724\times 10^{-2}\times {M_{w}}^{0.510}\;\left({\mathrm{cm}}^{3}∙g^{-1}\right)$$

It is commonly recognized that the exponent *α* serves as an indicator of polymer chain conformation in solution. Specifically, the *α* value below 0.5 suggested a highly flexible random coil in a good solvent; a range of 0.5 ≤ *α* < 0.8 corresponded to a typical random coil conformation; values between 0.8 ≤ *α* ≤ 2.0 indicated a rigid or rod-like chain (stiff chain conformation) (Guo et al., 2020). The index *α* value of 0.510 further proved that Q60E had a more compact shape of the polysaccharide chain and was a random coil conformation in aqueous solution.

### FT-IR analysis

The FT-IR spectrum of Q60E (Fig. 1C) displayed characteristic polysaccharide absorption bands. A broad and intense peak at 3363 cm^−1^ was ascribed to O—H stretching vibrations resulting from hydrogen bonding. Meanwhile, the second peak appearing at 2899 cm^−1^ corresponded to asymmetric stretching and bending vibrations of C—H bonds (Guo et al., 2020). The signal at 1636 cm^−1^ arose from the asymmetric tensile vibration of -COOH groups (Ke et al., 2021), while the peak at 1372 cm^−1^ suggested the possible existence of the -CH_2_ functional groups (Chen et al., 2025). In addition, the spectral range from 1200 cm^−1^ to 800 cm^−1^, known as the polysaccharide “fingerprint” region, exhibited multiple tensile vibrations, including C-C-O vibrations at 1080 cm^−1^, and C-OH bending at 1036 cm^−1^ (Abdin et al., 2025). Moreover, based on the “heterocephalic region” signals of glycosidic bonds, *β*-and *α*-linkage were exclusively identified at 897 cm^−1^ and 829 cm^−1^, respectively (Guo et al., 2020; Liu et al., 2018). These findings suggested that Q60E polysaccharide might contain both α and *β*-glycosidic bonds.

### Methylation analysis

The connection patterns of fragments in PMAA of Q60E were identified by mass spectral data and relative retention time in GC profiles (Fig. S1). The molar percentages for the methylated residues were derived from peak area ratios. The sugar residue compositions of Q60E (Table S3, Fig. 1D) mainly included →6)-Glc*p*-(1 → (47.23%), Glc*p*-(1 → (19.22%), →4,6)-Gal*p*-(1 → (9.93%), →3,6)-Glc*p*-(1 → (8.99%), →3)-Glc*p*-(1 → (5.30%), →4)-Glc*p*-(1 → (4.99%) and →3)-Man*p*-(1 → (4.34%), with the calculated molar ratio of 10.9: 4.4: 2.3: 2.1: 1.2:1.1:1.0, which was also in general agreement with the findings in the composition of monosaccharides.

### NMR and structural characterization

**Fig. 2:**
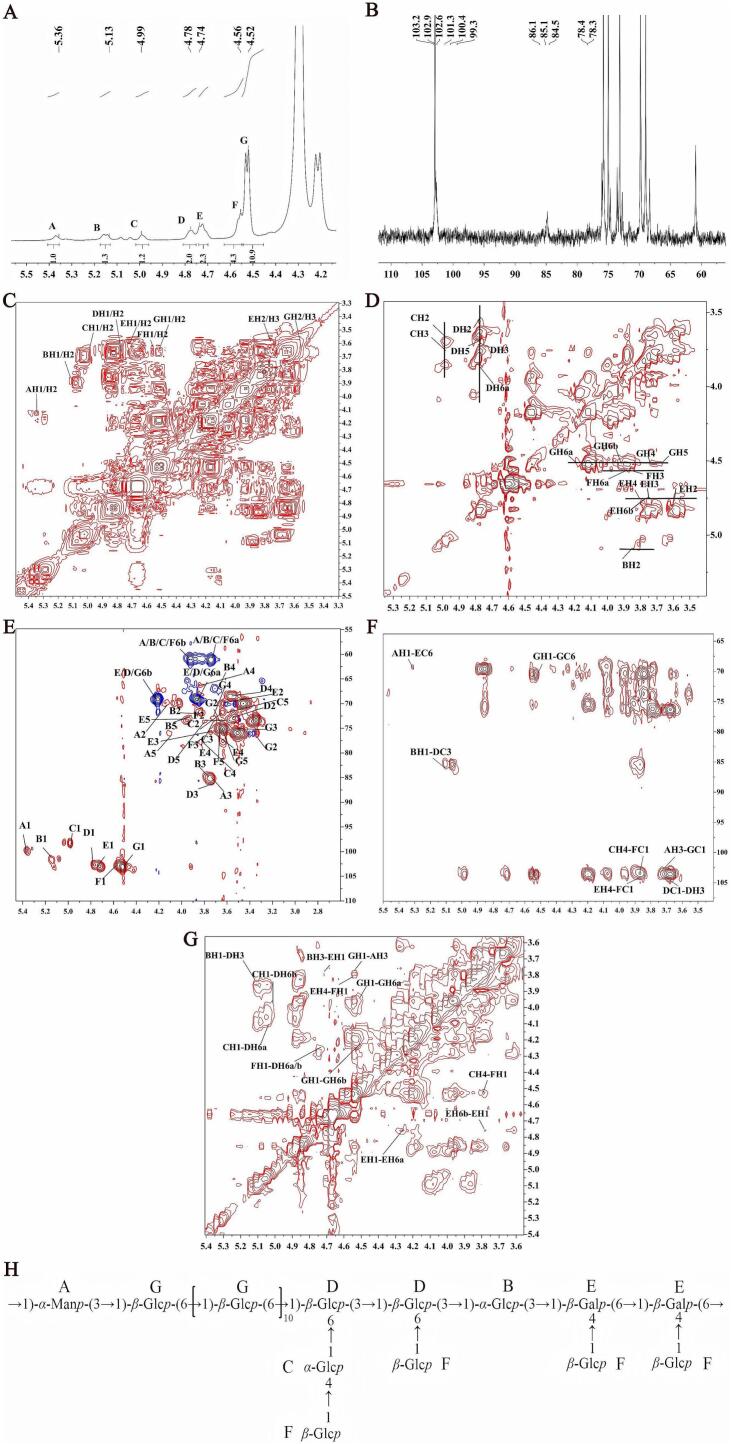
NMR spectra of Q60E: (A-G) ^1^H NMR, ^13^C NMR, COSY, TOCSY, HSQC, HMBC and NOESY spectra at 70 °C. H: the repeating structural unit of Q60E.

The results of NMR analysis (1D and 2D) further clarified the primary structure of Q60E. ^1^H NMR spectrum (Fig. 2A) showed seven different signal peaks at 5.36, 5.13, 4.99, 4.78, 4.74, 4.56 and 4.52 ppm, respectively, indicating that polysaccharide had both *α*- and *β-*configurations (Nandi et al., 2014; Shi et al., 2007). In addition, the peak area ratio of signal peaks in the ^1^H NMR spectrum was about 1.0: 1.3: 1.2: 2.0: 2.3: 4.3: 10.9, which was consistent with the molar ratio of glycosidic bonds in the methylation data. Furthermore, the ^13^C NMR spectrum (Fig. 2B) showed obvious signal peaks at 99.3–103.2 ppm, which further indicated that Q60E had *α*- and *β*-configurations (Nandi et al., 2014; Shi et al., 2007). The signal peak at about 85.1 ppm represented *O*-substituted C-3, the chemical shift of C-4 (78.4 and 78.3 ppm) signals represented the existence of (1 → 4) bond. According to the reduction of the chemical shift of anomeric proton, these seven glycosidic bonding parts were designated as A, B, C, D, E, F and G, respectively, as well as each of sugar residue A-G was further identified and analyzed by NMR spectrum, as detailed below:

The chemical shift of H-1 of sugar residue A was 5.36 ppm, and from the crossing peak signal of H-1 in COSY diagram (Fig. 2C), a correlation signal of 5.36/4.11 ppm was found, with 4.11 ppm attributed to H-2. The cross-peak correlation with H-2 (4.11/3.75 ppm) was analyzed to determine the H-3 chemical shift (3.75 ppm). The attribution of the chemical shift from H-2 to H-6 by this method could be confirmed by the TOCSY diagram of the chemical shift of H of *α*-sugar residue (Fig. 2D). The carbon signals were assigned based on their correlations with corresponding protons in the HSQC spectrum (Fig. 2E). The C and H chemical shifts of A were presented in Table 1. The molar ratio of monosaccharide composition, the proportion of glycosidic bonds in methylation and the downfield shift of the C-3 signal at 85.1 ppm further indicated that residue A was →3)-*α*-Man*p*-(1 → .

**Table 1:** The ^1^H NMR and ^13^C NMR chemical shifts of Q60E.

Glycosyl residues	H1/C1	H2/C2	H3/C3	H4/C4	H5/C5	H6a/C6	H6b
→3)-*α*-Man*p*-(1→	5.36	4.11	3.75	3.84	4.10	3.82	3.96
A	100.4	69.7	**85.1**	66.4	75.6	60.9	
→3)-*α*-Glc*p*-(1→	5.13	3.86	3.76	3.68	3.95	3.91	3.75
B	101.3	71.6	**84.5**	67.6	73.0	61.0	
→4)-*α*-Glc*p*-(1→	4.99	3.63	3.73	3.84	3.60	3.91	3.75
C	99.3	72.1	73.9	**78.3**	71.5	61.3	
→3,6)-*β*-Glc*p*-(1→	4.78	3.53	3.71	3.56	3.68	4.16	3.94
D	102.6	72.6	**86.1**	68.3	75.5	**69.8**	
→4,6)-*β*-Gal*p*-(1→	4.74	3.60	3.76	3.81	3.95	4.21	3.78
E	102.9	69.9	75.8	**78.4**	73.4	**69.4**	
*β*-Glc*p*-(1→	4.56	3.62	3.84	3.75	3.66	3.91	3.75
F	103.0	73.6	77.6	75.7	74.2	60.9	
→6)-*β*-Glc*p*-(1→	4.52	3.63	3.54	3.76	3.65	4.21	3.92
G	103.2	73.9	74.8	72.6	75.1	**69.8**	

The H-1 of sugar residue B appeared at 5.13 ppm, with its COSY correlation to H-2 (3.86 ppm) confirming this assignment. The H-3 and H-4 signals could be identified through cross-peaks in both COSY and TOCSY spectra. The chemical shifts of H-6a and H-6b were further determined by locating C-6 in HSQC spectrum, followed by the determination of the H-5 signal through the analysis of resonance cross signal of H-6a/b in COSY spectrum. Finally, the carbon chemical shifts in residue B were investigated using HSQC spectrum. The substitution chemical shift of C-3 (84.5 ppm) further confirmed that the sugar residue B was →3)-*α*-Glc*p*-(1 → .

Similarly, the H-1 of sugar residue C was identified at 4.99 ppm, with COSY correlations (Fig. 2C) confirming H-2 at 3.63 ppm. Subsequently, the H-3 position (3.73 ppm) was determined through H-2/H-3 coupling and further verified by TOCSY analysis (Fig. 2D). The HSQC spectrum (Fig. 2E) enabled the assignment of C-1 to C-6 signals based on their correlations with H-1 through H-6. As shown in Table 1, the notable downfield shift of C-4 (78.3 ppm) indicating *O*-4 substitution, and the sugar residue C could be determined as →4)-*α*-Glc*p*-(1 → .

The anomeric proton signal of D was observed at 4.78 ppm. Through analysis of COSY and TOCSY spectra, proton-proton correlations were established, enabling subsequent assignment of corresponding carbon chemical shifts in residue D *via* HSQC spectrum. The downfield chemical shifts observed for C-3 (86.1 ppm) and C-6 (69.8 ppm) established residue D as a → 3,6)-*β*-Glc*p*-(1 → (Liu et al., 2014).

In residue E, the anomeric proton resonance appeared at 4.74 ppm, with COSY and TOCSY spectral analysis enabling complete assignment of H-2 through H-6 protons. The results of chemical signals of C and H were shown in Table 1. Based on the C-4 (78.4 ppm) and C-6 (69.4 ppm) signals in the downfield shifts of methyl glucoside standard value (Agrawal, 1992), the sugar residue E was →4,6)-*β*-Gal*p*-(1 → .

The H-1 of residue F resonated at 4.56 ppm (H-1). Subsequent proton assignments (H-2 through H-6) were unambiguously established through comprehensive 2D NMR analysis, incorporating COSY, TOCSY and HSQC spectra. The chemical shift of sugar residue F was consistent with the standard glycosidic bond value (Agrawal, 1992), which indicated that the sugar residue F was *β*-Glc*p*-(1 → .

In sugar residue G, the H-1 signal (4.52 ppm) correlated with H-2 (3.63 ppm) in COSY spectrum, which in turn coupled with H-3 (3.54 ppm), completing the proton assignment. The C-6a/b signals were assigned from HSQC (Fig. 2E) using H-6a/b, followed by H-4/H-5 assignments (Table 1). The downfield-shifted C-6 (69.8 ppm) indicated *O*-6 substitution, and the sugar residue G could be determined as →6)-*β*-Glc*p*-(1 → .

The crossing peak signals of anomeric hydrogen and associated carbon in the sugar residue of Q60E component structure were confirmed by the crossing peak signals in HMBC spectrum (Fig. 2F), with the results presented in Table 2. The HMBC spectrum demonstrated significant relationships among sugar residues. Notably, H-3 (3.75 ppm) and H-1 (5.36 ppm) of A showed cross-peaks with both C-1 (103.2 ppm) of sugar residue G and C-6 (69.4 ppm) of sugar residue E, establishing A-G and A-E linkages. Additionally, the correlation between H-1 (5.13 ppm) of residue B and C-3 (86.1 ppm) of residue D confirmed the linkage between residues B and D. Further analysis revealed a crossing peak between H-4 (3.84 ppm) of C and C-1 (103.0 ppm) of F, indicating a correlation between residues C and F. Notably, an intramolecular crossing peak between H-3 (3.71 ppm) and C-1 (102.6 ppm) of sugar residue D suggested an internal linkage within residue D. Similarly, the H-1 (4.52 ppm) and C-6 (69.8 ppm) of sugar residue G showed an intramolecular connection, demonstrating its self-connectivity.

**Table 2:** Results of HMBC and NOESY spectrum analysis of residues in Q60E.

Sugar linkage	δ_*H*_	Observed connections in HMBC			NOESY contact proton		
		δ_*C*_	Residue	Atom	δ_*H*_	Residue	Atom
→3)-*α*-Man*p*-(1→	H-3/3.75	103.2	G	C-1	–	–	–
A	H-1/5.36	69.4	E	C-6	–	–	–
→3)-*α*-Glc*p*-(1→	H-1/5.13	86.1	D	C-3	3.71	D	H-3
B		–	–	–	–	–	–
→4)-*α*-Glc*p*-(1→	H-4/3.84	103.0	F	C-1	–	–	–
C	H-1/4.99	–	–	–	4.16/3.94	D	H-6a/b
→3,6)-*β*-Glc*p*-(1→	H-1/4.78	–	–	–	4.21/3.92	G	H-6a/b
D	H-3/3.71	102.6	D	C-1	–	–	–
→4,6)-*β*-Gal*p*-(1→	H-1/4.74	–	–	–	3.76 3.78/4.21	B E	H-3 H-6a/b
E	H-4/3.81	103.0	F	C-1	–	–	–
*β*-Glc*p*-(1→	H-1/4.56	–	–	–	3.84/3.81	C/E	H-4
F		–	–	–	4.16/3.94	D	H-6a/b
→6)-*β*-Glc*p*-(1→	H-1/4.52	69.8	G	C-6	4.21/3.92	G	H6a/b
G					3.75	A	H-3

This was then confirmed using the signals of the cross peaks of the correlated hydrogen and hydrogen in the NOESY spectrum (Fig. 2G and Table 2), and a cross peak was observed between the H-1 (5.13 ppm) of B and H-3 (3.71 ppm) of D, confirming the correlation between sugar residues B and D. A crossing peak between H-1 (4.99 ppm) of C and H-6a/H-6b (4.16/3.94 ppm) of D confirmed their linkage. Similarly, H-1 (4.78 ppm) of residue D correlated with H-6a/H-6b (4.21/3.92 ppm) of residue G, indicating an association between D and G. The H-1 (4.74 ppm) of residue E showed cross-peaks with both H-3 (3.76 ppm) of B and H-6a/H-6b (3.78/4.21 ppm) of E, suggesting E-B connectivity and an internal linkage within E. Additionally, H-1 (4.56 ppm) of residue F correlated with H-4 (3.84/3.81 ppm) of C and E, as well as H-6a/H-6b (4.16/3.94 ppm) of D, established the interrelation of F with C, E and D. Finally, a crossing peak between H-1 (4.52 ppm) of residue G and both its H-6a/H-6b (4.21/3.92 ppm) and H-3 (3.75 ppm) of residue A confirmed self-linkage in G and its connection to A. Based on the HMBC and NOESY correlations, the primary structure of the mannogalactoglucan (Q60E) repeat unit was determined to consist of →3)-*α*-Man*p*-(1→, →6)-*β*-Glc*p*-(1→, →3,6)-*β*-Glc*p*-(1 → and →3)-*α*-Glc*p*-(1 → glycosidic linkages forming the main chain, with side chains composed of →4)-*α*-Gal*p*-(1 → and terminal *β*-Glc*p*-(1 → linkages, as depicted in Fig. 2H.

The structures of polysaccharides currently identified in *H. erinaceus* primarily consisted of glucans, including *β*-(1 → 3)-glucose as the main chain, with *β*-glucose units attached at the *O*-6 position (Dong et al., 2006). Moreover, the heteropolysaccharide fraction identified in *H. erinaceus* comprised various structural types, including (1 → 6)-glucose and (1 → 4)-mannose linked as the main chain (Liao et al., 2020), *α*-(1 → 6)-glucose, *β*-(1 → 4)-galactose, and *α*-(1 → 3,6)-mannose as dominant chains (Liu et al., 2020b), as well as *β*-(1 → 6)-galactose and *β*-(1 → 2,6)-galactose forming the principal linkages (Wang et al., 2004). In this study, a polysaccharide fraction was isolated from the crude polysaccharides of SE pretreated *H. erinaceus* fruit bodies. It was identified as a mannogalactoglucan consisting of →3)-*α*-Man*p*-(1→, →6)-*β*-Glc*p*-(1→, →3,6)-*β*-Glc*p*-(1→, →3)-*α*-Glc*p*-(1 → and →4,6)-*β*-Gal*p*-(1 → as main chain, with branches including →4)-*α*-Glc*p*-(1 → and *β*-Glc*p* residues attached at specific positions as side chains, which further expanded the types of polysaccharide structures from *H. erinaceus*.

### Changes of physicochemical properties *in vitro* simulated digestion

#### Changes in reducing and total sugar contents

As shown in Supplementary Table S4, the reducing sugar and total sugar content of Q60E remained statistically unchanged (*p* > 0.05) throughout the *in vitro* oral digestion, which indicated that Q60E kept relatively stable during oral digestion phase. Next, the gastric digestion stage, most of the non-starch polysaccharides could be degraded due to the low pH level and enzymatic conditions presented in gastrointestinal tract, which also led to the break of glycosidic bonds in polysaccharide chains resulting in an increase in reducing sugar content (Wu et al., 2019). Additionally, the reducing and total sugar content exhibited no significant alteration during 4 h of gastric digestion (*p* > 0.05). Finally, the content of total sugar and reducing sugar remained unchanged after 6 h of intestinal digestion. This further showed that Q60E was resistant to catabolism by salivary and gastrointestinal fluids, and be directed to the colon for degradation by the GM.

#### Changes of molecular weights (*M*_*w*_)

**Fig. 3:**
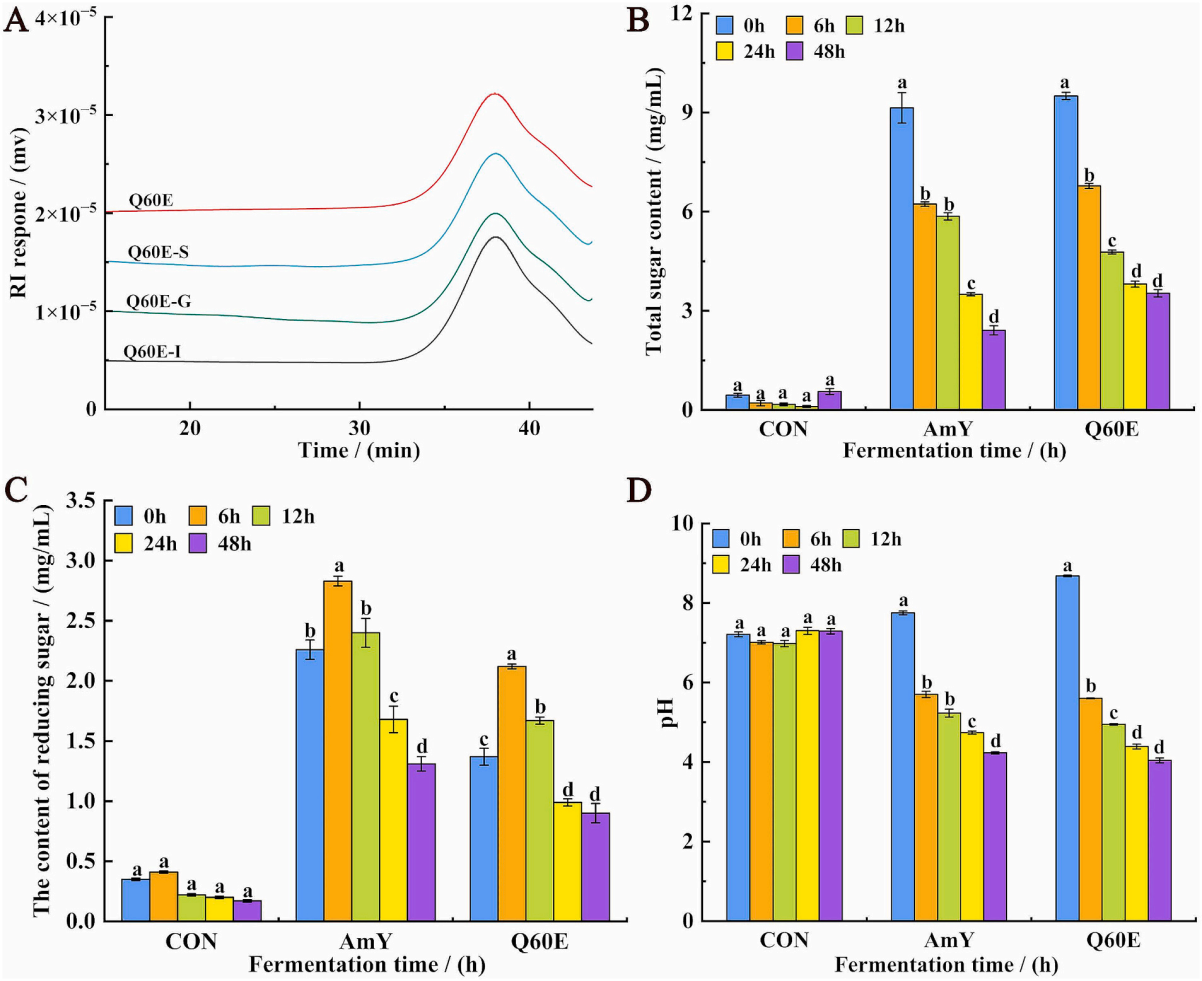
The *M*_*w*_ chromatogram simulating *in vitro* digestion (A), changes in reducing sugar content (B), total sugar content (C) and pH (D) during fermentation. Data are shown as mean ± SD (*n* = 3). Lowercase letters denote statistically significant differences (*p* < 0.05) among the same sample group at different time points, as determined by one-way ANOVA followed by Tukey's HSD post-hoc test. CON, blank control; AmY, basic carbon source control; Q60E, experimental group (0.2% Q60E + 0.8% starch). Q60E-S, Q60E-G and Q60E-I denoted the Q60E fraction after digestion in salivary, gastric and intestinal systems, respectively.

The *M*_*w*_ changes of Q60E at different stages during the three digestion processes *in vitro* were investigated. Analysis of the *M*_*w*_ distribution revealed that Q60E remained largely consistent throughout the simulated digestion phases, as evidenced by the stable elution profiles ranging from 33 to 44 min in Fig. 3A. The *M*_*w*_ of Q60E in saliva- stomach -small intestine was 8.894 × 10^4^ g/mol, 8.422 × 10^4^ g/mol and 8.381 × 10^4^ g/mol, respectively, and there was no obvious change (*p* > 0.05). This observation was consistent with the trends in reducing sugar and total sugar content during the digestion process (Zhang et al., 2023), further confirming that the majority of polysaccharide components remained stable throughout the *in vitro* simulated digestion.

### Analysis of fermentation results *in vitro*

#### Changes of physicochemical properties of fermentation products

In the process of colon fermentation, the changes of chemical indexes such as total carbohydrate content, reducing sugar and pH value could serve as indicators of the employing and degradation of the polysaccharide by GM (Gao et al., 2024). The data showed that the content of most carbohydrates in the Q60E decreased from 9.50 mg/mL at 0 h to 3.53 mg/mL at 48 h (Fig. 3B), suggesting that the polysaccharide could be further consumed and utilized by the GM.

On the other hand, indigestible polysaccharide chains could be utilized and degraded by GM in colon to produce reducing sugar as a carbon source for the microorganisms (Ding et al., 2019). Therefore, the correlation between glycosidic bond breaking and the proliferation of the GM could be reflected by the content of reducing sugar. During *in vitro* fecal fermentation, the reducing sugar content of indigestible polysaccharide (Q60E) increased significantly (*p* < 0.05) to 2.12 ± 0.02 mg/mL after 6 h, followed by a gradual decline (Fig. 3C), consistent with previously reported results (Zhang et al., 2023), suggesting that glycosidic bond cleavage in Q60E predominates during the initial 6 h fermentation phase. Subsequently, the released fermentation products were further metabolized by the GM during the subsequent fermentation process duration ranging from 6 to 48 h.

In addition, a significant reduction (*p* < 0.05) in pH values was observed in both the AmY and Q60E carbon source groups, reaching 4.23 and 4.04, respectively, after 48 h of fermentation (Fig. 3D). Low pH levels in the intestine could modulate the GM composition by enriching beneficial bacteria while suppressing detrimental ones (Shoukat & Sorrentino, 2021).

#### Effects in human GM composition

**Fig. 4:**
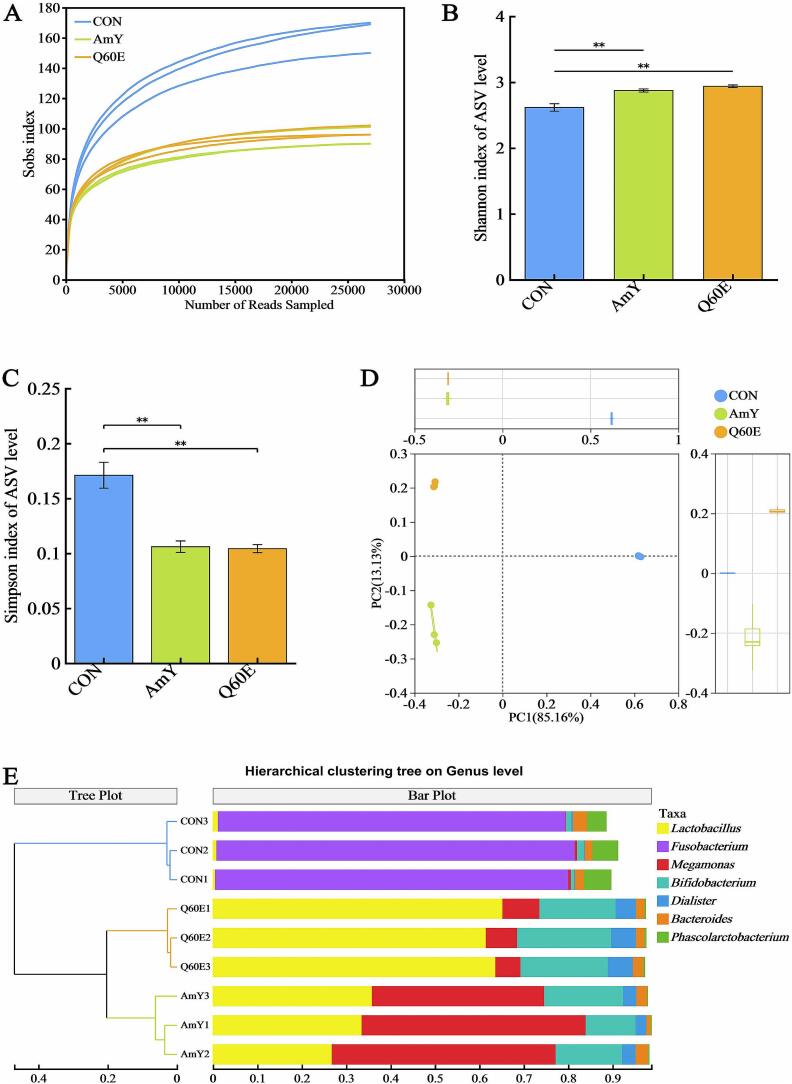
Effect of *in vitro* fermentation on GM *α*-diversity: Sobs index (A), Shannon index (B) and Simpson index (C). *β*-diversity analysis comprises principal co-ordination analysis (PCoA) (D) and Hierarchical clustering analysis (E) based on UPGMA algorithm. CON, blank group; AmY, 1% basic carbon source group; Q60E, experimental group (0.2% Q60E + 0.8% starch). **, 0.001 < *p* ≤ 0.01.

In this study, the *α*-diversity (Sobs, Shannon and Simpson indices) and *β*-diversity (PCoA and hierarchical cluster analysis) of the species in the GM were explored by employing 16S rRNA genes sequencing technology, which primarily reflect compositional shifts rather than absolute changes in bacterial load. Fig. 4A demonstrated that the Sobs sparsity curves stabilized with increasing number of reads, indicating that reasonable sequencing data were obtained. Furthermore, the supplementation of Q60E polysaccharide led to greater GM richness than that observed in the basic carbon source AmY group, which was consistent with previous investigations (Chen et al., 2025). Shannon and Simpson indices were positively and negatively correlated with the diversity of GM, respectively. Therefore, as illustrated in Fig. 4B and C, the carbon source group with AmY and Q60E increased the diversity of GM compared to the CON group. These results showed that after 48 h of fermentation, supplementing starch with Q60E further increased community richness and diversity compared with the AmY group. This additive benefit can be attributed to the ability of the GM to utilize Q60E as an additional carbon source, thereby enhancing overall microbial diversity and potentially promoting the growth of specific beneficial genera. Principal coordinate analysis (PCoA) based on ASV relative abundance revealed distinct microbial community shifts among the CON, AmY and Q60E groups (Fig. 4D). The first two principal coordinates (PC1; 85.16% and PC2; 13.13%) explained 98.29% of the total variation. Notably, both AmY and Q60E clustered distinctly from CON, indicating that Q60E markedly influenced microbial community composition. In addition, hierarchical cluster analysis using the UPGMA algorithm was performed to construct a dendrogram, visually representing community distribution differences among the three groups. The resulting clustering pattern (Fig. 4E) aligned with the PCoA results, further confirming that both Q60E and AmY significantly influenced microbial community composition.

**Fig. 5:**
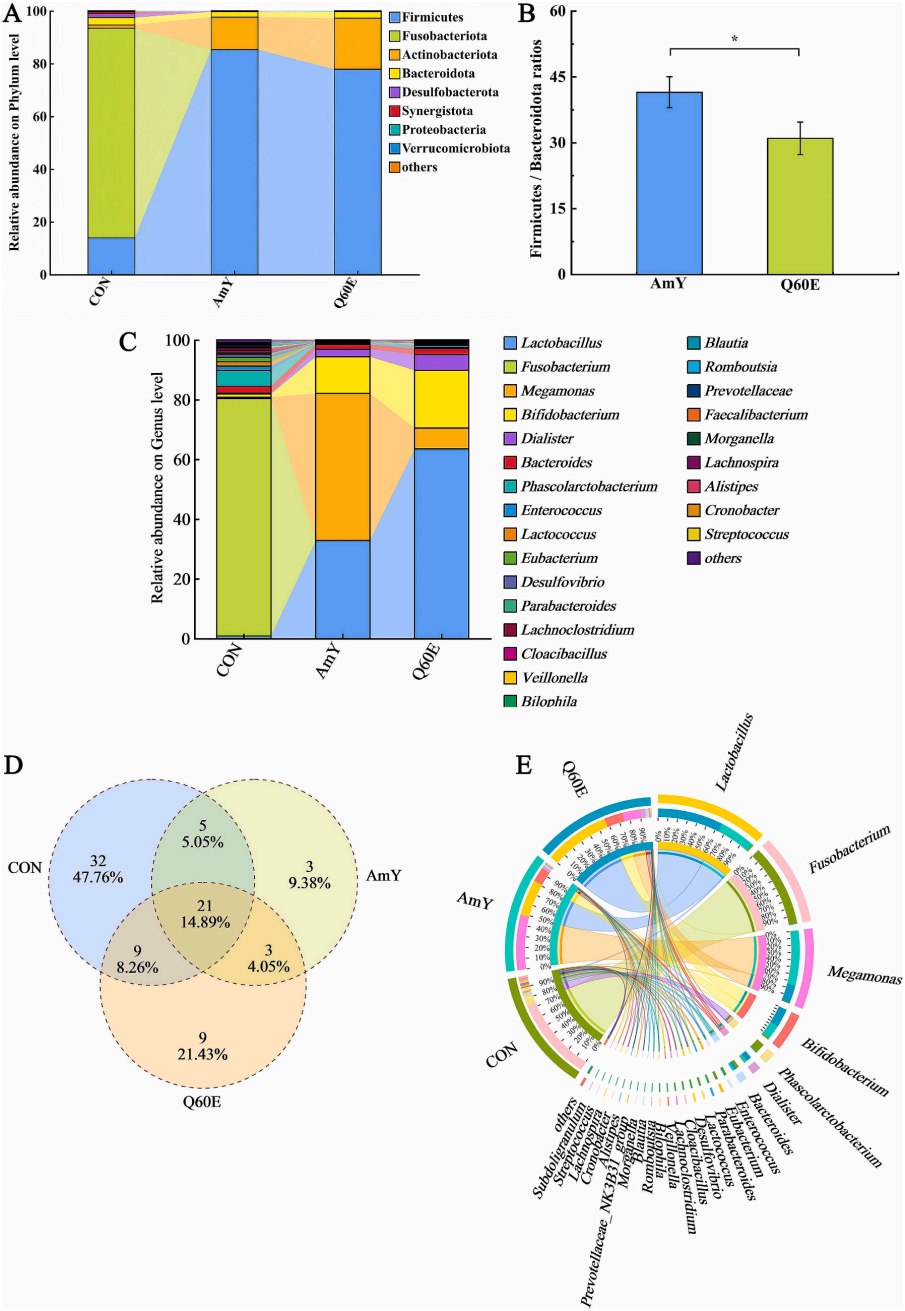
GM modulation by Q60E after 48 h fermentation. (A) Phylum-level composition; (B) Firmicutes/Bacteroidota (F/B) ratio (* *p* < 0.05, Q60E *vs* AmY), as determined by ANOVA followed by Tukey's HSD post-hoc test; (C) Heatmap of top 20 genera clustered by abundance; (D) Venn diagram of ASVs; (E) Circos diagram showing phylum - genus associations. Data represent mean ± SD (*n* = 3). CON, blank control; AmY, basic carbon source control; Q60E, experimental group (0.2% Q60E + 0.8% starch).

At the phylum level, the predominant bacterial communities included Actinobacteriota, Firmicutes, Fusobacteriota and Bacteroidota (Fig. 5A). The relative abundance of Firmicutes was significantly higher in the AmY (85.26%) and Q60E groups (77.82%) compared to the CON group (13.90%), while studies have shown that some Firmicutes in the intestine could play a positive regulatory role in host health by fermenting indigestible carbohydrates to produce some metabolites such as SCFAs (Xu et al., 2019). In addition, both carbon source groups significantly reduced the abundance of Fusobacteriota, which was usually regarded as a conditionally pathogenic bacterium that could be increased by nutritional deficiencies and was associated with cancer (Brennan & Garrett, 2019), compared to the CON group (79.51%). Additionally, the Q60E group exhibited a substantially lower Firmicutes to Bacteroidota (F/B) ratio than the AmY group (*p* < 0.05, Fig. 5B). This reduction was consistent with some reports linking a lower F/B ratio to reduced adiposity (Abdin et al., 2025; Le Chatelier et al., 2013). However, the broader relationship between the F/B ratio and metabolic health was known to vary depending on host metabolic status, dietary background and GM community structure. Compared with CON group, AmY and Q60E groups also significantly increased the abundance of Actinobacteriota. The Actinobacteriota mainly contained beneficial bacteria such as *Lactobacillus* and *Bifidobacterium*, which could produce lactic acid, acetic acid and other metabolites to enhance human intestinal immunity (Zhang et al., 2023).

The three groups exhibited distinct GM distributions at the genus level, as illustrated in Fig. 5C. *Lactobacillus*, *Megamonas*, *Bifidobacterium*, *Bacteroides*, *Dialister* and *Phascolarctobacterium* constituted the vast majority of the carbon source groups (AmY and Q60E). *Bacteroides* could degrade indigestible polysaccharides by encoding most carbohydrate active digestive enzymes and utilize them to produce SCFAs (Chen et al., 2024). In addition, *Phascolarctobacterium* could produce mainly acetic and propionic acids, which was also showed a positive correlation with the improvement of the mood of the organism (Ding et al., 2019). The AmY group demonstrated a substantial increase in *Megamonas* abundance (49.27%) in comparison to the CON group, which was capable of degrading and using starch to sustain its own proliferation. Furthermore, its expansion competitively inhibited the growth of harmful bacteria by limiting carbohydrate availability, while primarily producing acetic and propionic acids as key metabolites (Zhou et al., 2022). Compared with the CON and AmY groups, the addition of polysaccharide Q60E focused on increasing the abundance of *Lactobacillus*, *Bifidobacterium* and *Dialister*, aligning with the effects illustrated in the Circos diagram (Fig. 5E) on the GM. Among them, *Lactobacillus* could increase the production of beneficial metabolites and reduce the risk of organism being infected by pathogens through promoting carbohydrate degradation and inhibiting the proliferation of harmful microorganisms (Guan et al., 2025; Zhang et al., 2018). *Bifidobacterium*, a key beneficial intestinal microbe, primarily produced acetic and propionic acids through fermentation. Its metabolites exhibited significant physiological benefits, including enhanced intestinal immunity and anti-tumor properties (Di Gioia, Aloisio, Mazzola and Biavati, 2014). *Dialister* was a core bacterium of the human gut, belonging to the phylum Firmicutes, capable of producing acetic and propionic acids as well as histamine through the digestion of nutrients (Morio et al., 2007). It is further indicated that the different monosaccharide composition and glycosidic bond connection in polysaccharides were the factors that affect the composition of the GM (Wang et al., 2019). The Venn diagram (Fig. 5D) showed the number of unique and common species in three different groups, where the overlapping part indicated that the number of common species in the three sample groups was 21. Compared with the AmY group, Q60E group exhibited an increase in the number of unique GM, achieving 9 ASV counts.

**Fig. 6:**
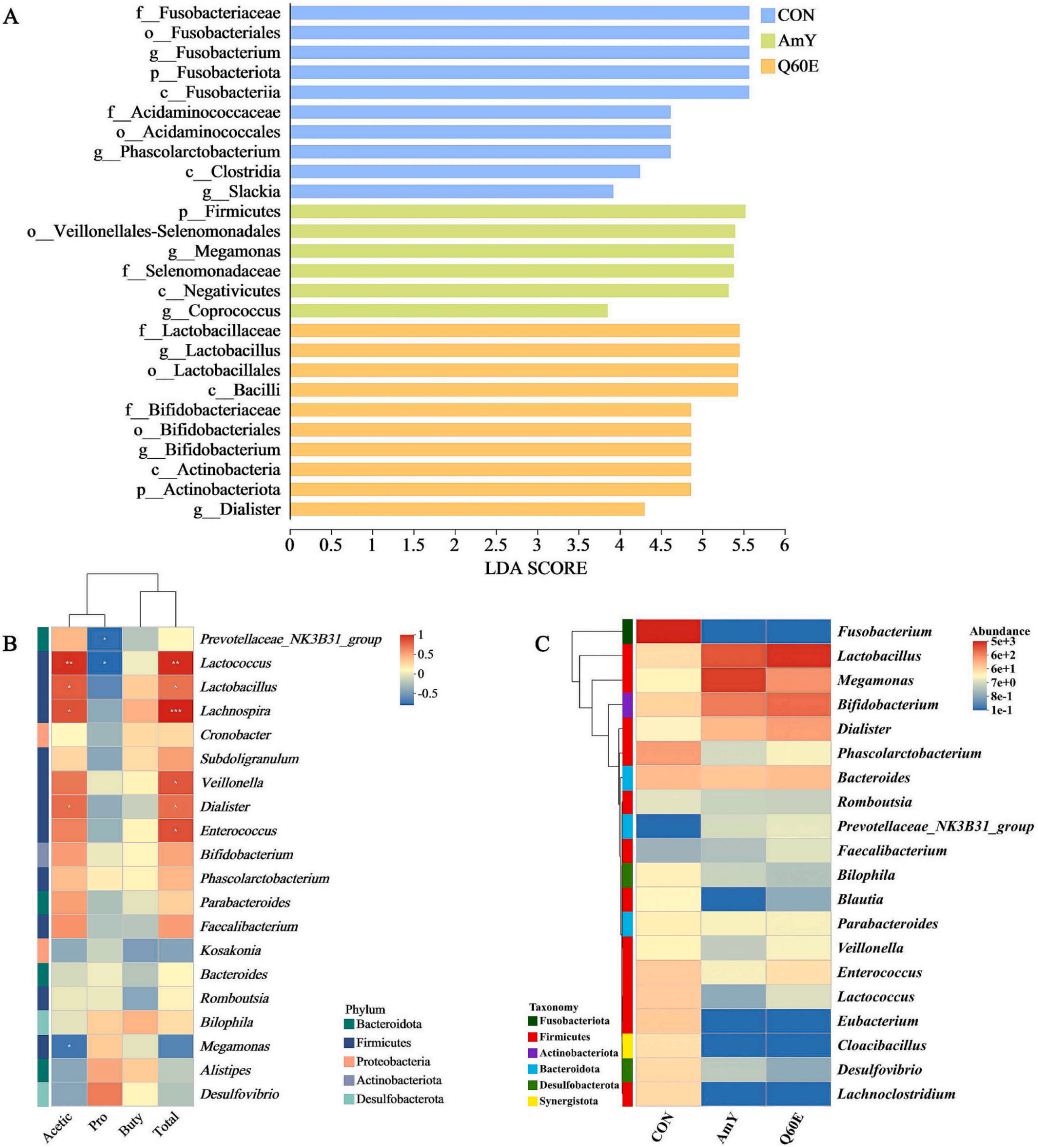
Linear discriminant analysis (LDA > 3) (A); the Heatmap visualization of GM-SCFA correlations (B); and the genus-level abundance heatmap (C). Statistical significance was denoted as: * (0.01 < *p* ≤ 0.05), ** (0.001 < *p* ≤ 0.01) and *** (*p* ≤ 0.001) in Fig. 6C, as determined by Kruskal-Wallis test followed by Dunn's multiple comparison test with FDR correction. CON, blank control group; AmY, basic carbon source control group; Q60E, experimental group (0.2% Q60E + 0.8% starch).

Tests of variance based on the linear discriminant analysis effect size (LEfSe) research (from phylum to genus) to analyze variance species at multiple levels and measure the magnitude of species effects on variance effects using LDA values to determine the changes in key GM species affected by the addition of carbon sources. As shown in Fig. S2, the dominant flora in the Q60E group comprised 18 distinct species, including *p_Actinobacteriota*, *o_Bifidobacteriales*, *f_Lactobacillaceae*, *g_Dialister*, *g_Lachnospira* and *g_Prevotellaceae_NK3B31_group*, while the dominant flora in the AmY group included *p_Firmicutes*, *c_Negativicutes*, *g_Megamonas*, *f_Selenomonadaceae* and *g_Coprococcus* with 6 differential species. *Lachnospira* affect host mucosal immune cells and intestinal cells by producing SCFAs and promoting colonization resistance against intestinal pathogens (Sorbara et al., 2020). It is reported that *Prevotellaceae* played an important role in carbohydrate consumption and host metabolism (Si et al., 2017). The higher LDA value (Fig. 6A) indicated the robustness of the differential impact of this sample on flora species, which was consistent with LEfSe. Compared with CON group, the Q60E component inhibited the propagation of the pathogen *Fusobacteriaceae* to a certain extent. All these results indicated that supplementation with the Q60E polysaccharide fraction effectively increased both the abundance and diversity of beneficial GM, demonstrating excellent prebiotic potential.

The heatmap analysis (Fig. 6C) visualized the relative abundance distribution of the top 20 genera across experimental groups CON, AmY and Q60E, with darker colors indicating the proportion of bacteria containing higher abundance. Notably, Q60E significantly enriched (*p* < 0.05) beneficial bacterial genera such as *Lactobacillus*, *Bifidobacterium* and *Bacteroides* compared to the AmY. This finding aligns with a previous study demonstrating that mannogalactoglucan could promote the proliferation of beneficial bacteria (*Lactobacillus*) and enhance the yield of SCFAs (Chen et al., 2022). Additionally, the monosaccharide composition of polysaccharides might influence fecal GM composition. Generally, the addition of Q60E was superior to the AmY group in promoting the population and diversity of beneficial bacteria.

#### Potential relationship between SCFAs production and GM

**Fig. 7:**
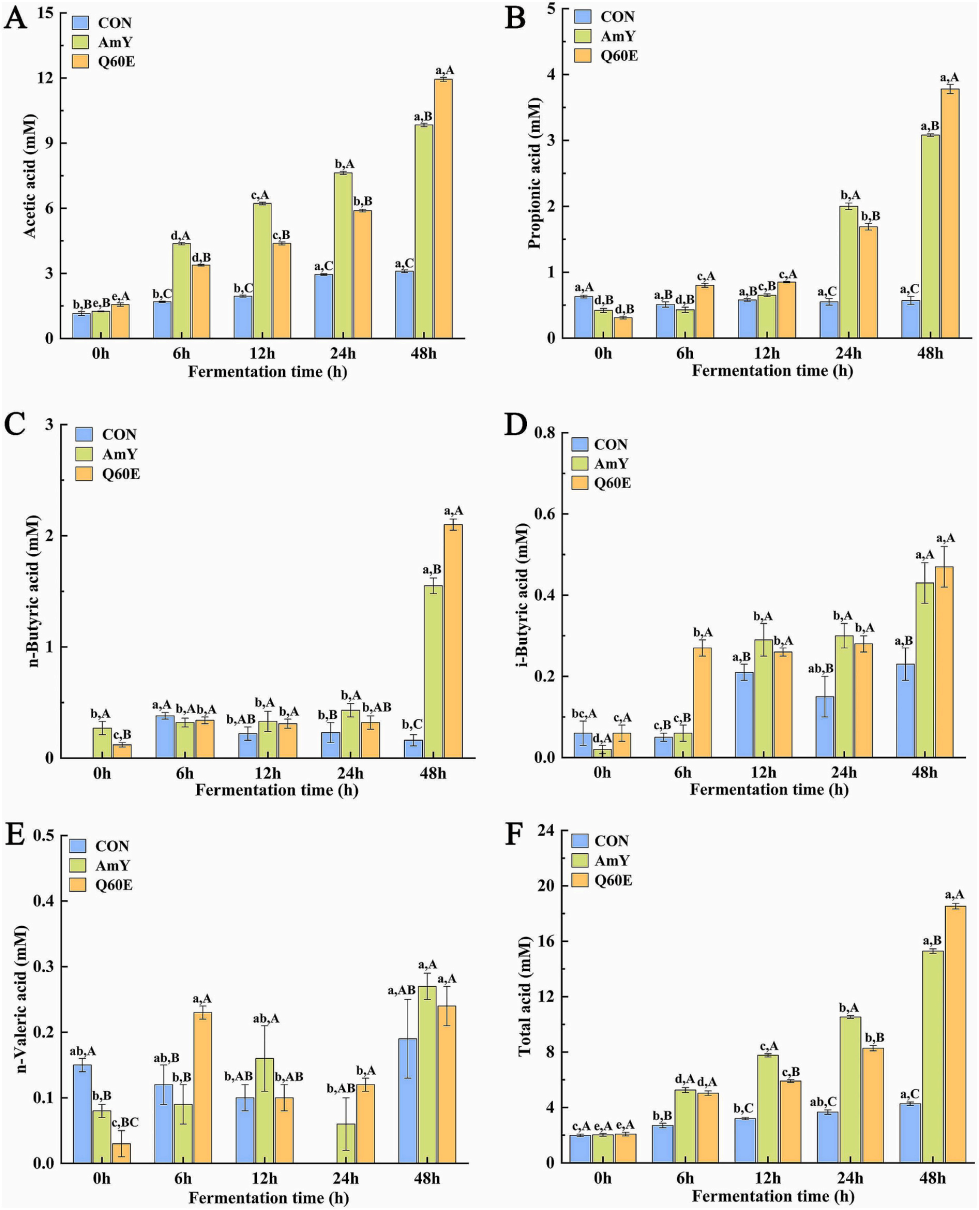
The changes in Acetic (A), Propionic (B), n-Butyric (C), i-Butyric (D), n-Valeric (E) acids and Total acids (F) during fermentation, respectively. Results are expressed as mean ± SD (*n* = 3), different lower-case letters represent significant difference across time points within each group (*p* < 0.05), while different upper-case letters indicate significant difference between-groups at same time point (*p* < 0.05), as determined by ANOVA followed by Tukey's test.

The SCFAs for each time period were shown in Fig. 7, and the total SCFAs of AmY and Q60E accumulated swiftly within 6–48 h of simulated fermentation, attaining 15.29 mmol/L for the former and 18.53 mmol/L for the latter. This further demonstrated that the Q60E fraction added to the fermentation process could be digested and employed by the GM. Additionally, the polysaccharide with a low *M*_*w*_ was also more easily fermented, resulting in an increase in the yield of SCFAs (Li et al., 2022; Lai et al., 2024). The yield of acetic and propionic acids in Q60E polysaccharide group was substantially increased (*p* < 0.05) in comparison to AmY, reaching 11.94 mmol/L and 3.78 mmol/L, respectively, this result was consistent with the previous research that mannogalactoglucan could improve the production of acetic and propionic acids (Chen et al., 2022). The literature has reported that transgenics were able to more readily utilize polysaccharides with a lower *M*_*w*_ to generate metabolites including acetate and propionic acid (Xie et al., 2020). Additionally, acetic acid had the potential to be metabolized in the kidneys and absorbed by body tissues for energy utilization (Frost et al., 2014). Propionic acid could inhibit the synthesis of cholesterol and significantly influence glucose metabolism and energy equilibrium (Ding et al., 2019). Finally, the 16S rRNA sequencing and SCFAs content demonstrated that Q60E significantly reshaped the diversity and composition of the GM. The results of various SCFAs analysis after 48 h of fermentation further showed that Q60E generated higher levels of acetic acid, propionic acid and total acid, which corroborated previous findings (Chen et al., 2025).

Exploring the correlation coefficients between the top 20 dominant microorganism groups and SCFAs based on the genus level of GM, allowed for the identification of a certain microorganism that might be the key species influencing SCFAs. Through the comparative analysis of Fig. 6B and Fig. S2, it is speculated that the Q60E might promote the acetic acid generation by raising the abundance of *Lactobacillus* and *Lachnospira*. In addition, the rise in the relative abundance of *Lachnospira* in Q60E coincided with an enhancement in total acids. Acetic acid was the main end product of *Lactobacillus* fermentation, which could control appetite and resist pathogens through the blood-brain barrier and central homeostatic mechanisms (Ferreira-Lazarte et al., 2019). *Lachnospira*, a member of the phylum Firmicutes, was present in the intestinal system of most healthy individuals and is considered to be potentially positive bacterium involved in the metabolism of various carbohydrates, which in turn produced acetic and butyric acids to provide the host with a major source of energy (Sorbara et al., 2020). The enrichment of *Lachnospira* and *Bifidobacterium* following Q60E fermentation correlated with elevated SCFAs production, suggesting that the specific glycosidic linkage pattern may selectively stimulate SCFA-producing taxa.

This evidence suggested that Q60E polysaccharide could play a prebiotic role by increasing the production of beneficial bacteria and metabolites (acetic and total acids), which might have beneficial effects on organismal health. Although the Q60E fraction positively influenced the intestinal health by regulating GM-mediated fermentation products, further long-term *in vivo* animal experiments were required to validate the underlying mechanisms involving beneficial flora and differential metabolites.

## Conclusions

In this study, a mannogalactoglucan (Q60E) was isolated from the crude polysaccharides of *H. erinaceus* following SE pretreatment. Structural characterization revealed that its main chain was composed of *α*-(1 → 3)-Man*p*, *β*-(1 → 4,6)-Gal*p*, *β*-(1 → 6)-Glc*p*, *β*-(1 → 3,6)-Glc*p* and *α*-(1 → 3)-Glc*p* residues. The side chains consisted of *α*-(1 → 4)-Glc*p* and *β*-Glc*p* residues attached at the *O*-6 position of *β*-(1 → 3,6)-Glc*p*, with terminal *β*-Glc*p* units branching from both the *O*-6 position of *β*-(1 → 3,6)-Glc*p* and the *O*-6 position of *β*-(1 → 4,6)-Gal*p*. The polysaccharide exhibited resistance to degradation under simulated oral-gastric-intestinal digestion and was efficiently utilized by GM, indicating its prebiotic potential linked to SCFAs production and GM composition modulation. Compared with starch alone, Q60E supplementation (in the presence of starch) significantly altered the microbiome. Specifically, Q60E intervention markedly reshaped the GM structure, leading to a significant increase in Actinobacteriota and Bacteroidota (*p* < 0.05) and a pronounced reduction in Fusobacteriota. At the genus level, it selectively enriched beneficial bacteria such as *Lactobacillus*, *Bifidobacterium* and *Lachnospira*, which were correlated with elevated levels of acetic acid and total SCFAs. These findings suggest potential microbiota-modulating and SCFA-promoting effects of this mannogalactoglucan. Building upon these insights, future *in vivo* studies will be undertaken to confirm its regulatory activity and to further assess its health implications. This work provides a foundation for the development of SE-pretreated *H. erinaceus* polysaccharides as functional ingredients targeting gut health.

## CRediT authorship contribution statement

**Shuang Chen:** Writing – original draft, Formal analysis, Conceptualization. **Qing Yan:** Investigation, Data curation. **Liping Liu:** Methodology, Investigation, Formal analysis. **Jie Feng:** Methodology, Investigation. **Qing Kong:** Investigation. **Jingsong Zhang:** Methodology, Investigation, Formal analysis. **Jie Geng:** Investigation. **Jinyan Wang:** Methodology, Investigation. **Qingbin Guo:** Writing – review & editing, Validation, Conceptualization. **Yanfang Liu:** Writing – review & editing, Supervision, Funding acquisition, Conceptualization.

## Declaration of competing interest

The authors declare that they have no known competing financial interests or personal relationships that could have appeared to influence the work reported in this paper.

## Data Availability

Data will be made available on request.
