# Supplementary material for: A mannogalactoglucan from steam-exploded Hericium erinaceus: structural elucidation, digestion resistance and gut microbiota-modulating prebiotic activity
Source: Food Chem X. 2026 Jul 21;38:104233. doi: 10.1016/j.fochx.2026.104233 (PMC13445368; doi:10.1016/j.fochx.2026.104233)
Supplement: Supplementary file 1 [file mmc1.docx]

**Supplementary information**

**A mannogalactoglucan from steam-exploded *Hericium erinaceus*: Structural elucidation, digestion resistance and gut microbiota-modulating prebiotic activity**

Shuang Chen^a,b^, Qing Yan^a,b^, Liping Liu^a^, Jie Feng^a^, Qing Kong^c^, Jingsong Zhang^a^, Jie Geng^a^, Jinyan Wang^a^, Qingbin Guo^b*^, Yanfang Liu^a*^

*^a^ Institute of Edible Fungi, Shanghai Academy of Agricultural Sciences, National Engineering Research Center of Edible Fungi, Key Laboratory of Edible Fungi Resources and Utilization (South), Ministry of Agriculture and Rural Affairs, Shanghai 201403, China*

*^b^ State Key Laboratory of Food Nutrition and Safety, College of Food Science and Engineering, Tianjin University of Science and Technology, Tianjin, 300457, China*

*^c^ Shandong Medicine Technician College, Taian, Shandong, 271000, China*

^*^ Corresponding authors (Y. F. Liu). Tel: +86 21-37195727. E-mail: aliu-1980@163.com. (Q. B. Guo). E-mail: guoqingbin008322@tust.edu.cn.

**Supplementary data**

Figure. S1. Individual mass spectrum of major sugar residues in Q60E. Glc*p*-(1→ (A), →3)-Glc*p*-(1→ (B), →4)-Glc*p*-(1→ (C), →6)-Glc*p*-(1→ (D), →3)-Man*p*-(1→ (E), →4,6)-Gal*p*-(1→ (F), →3,6)-Glc*p*-(1→ (G). Q60E, the experimental group (the polysaccharides from *H. erinaceus* fruiting bodies pretreated by steam explosion).

Figure. S2. Linear discriminant analysis effect size (LEfSe) analysis revealing differential taxa from phylum to genus level among groups (n=3). CON, blank control group; AmY, basic carbon source control group; Q60E, experimental group (0.2% Q60E + 0.8% starch).

Table S1 Yield, chemical components and monosaccharide compositions of Q60E

| Index | Percentage (%) |
| --- | --- |
| Yield | 19.21±1.35 |
| Component content | |
| Total sugar | 89.71±0.50 |
| Protein | 5.37±0.34 |
| Monosaccharide molar percent | |
| Galactose | 8.35 |
| Glucose | 87.14 |
| Mannose | 4.51 |

Q60E, the experimental group (the polysaccharides from *H. erinaceus* fruiting bodies pretreated by steam explosion).

Table S2 Macromolecular and conformational characterizations of Q60E

| Sample  name | *M_w_*  (g/mol) | *M_n_*  (g/mol) | Polydispersity  (PDI) | *R_g_*  (nm) | *R_h_*  (nm) | [*η*]  (mL/g) |
| --- | --- | --- | --- | --- | --- | --- |
| Q60E | 8.89×10^4^ | 5.37×10^4^ | 1.66 | 15.80 | 9.26 | 15.81 |

Q60E, the experimental group (the polysaccharides from *H. erinaceus* fruiting bodies pretreated by steam explosion).

Table S3 Methylation analysis data for Q60E

| Methylated sugar | Type of linkage | Molar ratio (%) | Major mass fragments (m/z) |
| --- | --- | --- | --- |
| 2,3,4,6-Me_4_-Glc | Glc*p*-(1→ | 19.22 | 43,45,71,87,102,118,129,145,161,162,175,205 |
| 2,4,6-Me_3_-Glc | →3)-Glc*p*-(1→ | 5.30 | 43,45,71,87,101,118,129,161,174,202,218,234,277 |
| 2,3,6-Me_3_-Glc | →4)-Glc*p*-(1→ | 4.99 | 43,59,71,87,102,113,118,129,142,162,173,203,233 |
| 2,3,4-Me_3_-Glc | →6)-Glc*p*-(1→ | 47.23 | 43,71,87,99,102,118,129,143,159,162,173,189,233 |
| 2,4,6-Me_3_-Man | →3)-Man*p*-(1→ | 4.34 | 43,59,71,87,101,118,129,143,161,174,190,202,217,234 |
| 2,3-Me_2_-Gal | →4,6)-Gal*p*-(1→ | 9.93 | 43,59,85,102,118,127,142,159,162,187,201,261,305 |
| 2,4-Me_2_-Glc | →3,6)-Glc*p*-(1→ | 8.99 | 43,59,74,87,118,129,139,160,174,189,234,245,305 |

Table S4 Changes in reducing sugar and total sugar of Q60E during *in vitro* digestion

| Process | Time | Reducing sugar (mg/mL) | Total sugar (mg/mL) |
| --- | --- | --- | --- |
| Saliva digestion | 0 min | 0.006±0.001^a^ | 1.846±0.016^a^ |
|  | 2 min | 0.009±0.003^a^ | 2.098±0.008^a^ |
| Gastric digestion | 0 h | 0.015±0.004^a^ | 0.957±0.007^b^ |
|  | 2 h | 0.013±0.002^a^ | 1.109±0.009^b^ |
|  | 4 h | 0.012±0.005^a^ | 1.078±0.010^b^ |
| Intestinal digestion | 0 h | 0.009±0.002^a^ | 0.551±0.005^c^ |
|  | 2 h | 0.007±0.003^a^ | 0.506±0.006^c^ |
|  | 4 h | 0.010±0.004^a^ | 0.516±0.021^c^ |
|  | 6 h | 0.008±0.003^a^ | 0.622±0.019^c^ |

Data are expressed as mean ± standard deviation (SD) (n=3), and lowercase letter means significant differences in different digestion stages at different time points (*p* < 0.05) by a Tukey test. Q60E, the experimental group (the polysaccharides from *H. erinaceus* fruiting bodies pretreated by steam explosion).
